# Entanglement and quantum coherence of two YIG spheres in a hybrid Laguerre–Gaussian cavity optomechanics

**DOI:** 10.1038/s41598-024-61670-7

**Published:** 2024-05-16

**Authors:** Abdelkader Hidki, Jia-Xin Peng, S. K. Singh, M. Khalid, M. Asjad

**Affiliations:** 1 LPTHE, Department of Physics, Faculty of Sciences, Ibn Zohr University, Agadir, Morocco; 2 School of Physics and Electronic Electrical Engineering, Huaiyin Normal University, Huaian, 223300 China; 3 Process Systems Engineering Centre (PROSPECT), Research Institute of Sustainable Environment (RISE), School of Chemical and Energy Engineering, Universiti Teknologi Malaysia, 81310 Johor Bahru, Malaysia; 4 Graphene and Advanced 2D Materials Research Group (GAMRG), School of Engineering and Technology, Sunway University, Petaling Jaya, Selangor Malaysia; 5 Sunway Centre for Electrochemical Energy and Sustainable Technology (SCEEST), School of Engineering and Technology, Sunway University, No. 5, Jalan University, Bandar Sunway, 47500 Petaling Jaya, Selangor Malaysia; 6 Centre of Research Impact and Outcome, Chitkara University, Chandigarh, Punjab 140401 India; 7 Department of Applied Mathematics and Sciences, Khalifa University, 127788 Abu Dhabi, UAE

**Keywords:** Quantum information, Quantum optics, Physics

## Abstract

We theoretically investigate continuous variable entanglement and macroscopic quantum coherence in the hybrid L–G rotational cavity optomechanical system containing two YIG spheres. In this system, a single L–G cavity mode and both magnon modes (which are due to the collective excitation of spins in two YIG spheres) are coupled through the magnetic dipole interaction whereas the L–G cavity mode can also exchange orbital angular momentum (OAM) with the rotating mirror (RM). We study in detail the effects of various physical parameters like cavity and both magnon detunings, environment temperature, optorotational and magnon coupling strengths on the bipartite entanglement and the macroscopic quantum coherence as well. We also explore parameter regimes to achieve maximum values for both of these quantum correlations. We also observed that the parameters regime for achieving maximum bipartite entanglement is completely different from macroscopic quantum coherence. So, our present study shall provide a method to control various nonclassical quantum correlations of macroscopic objects in the hybrid L–G rotational cavity optomechanical system and have potential applications in quantum sensing, quantum meteorology, and quantum information science.

## Introduction

Cavity Optomechanics (COM) explores the interaction between the electromagnetic field and the mechanical motion through the radiation pressure^1^ and in the last decades, a significant progresses achieved in this area emerging of research^2–6^. It has also made several advances in the modern era of quantum technology such as ultrahigh-precision measurement^7^, gravitation-wave detection^8^, quantum entanglement^9–15^, macroscopic quantum coherence^16,17^, optomechanically induced transparency/absorption phenomena (OMIT/OMIA) and normal mode splitting^18–22^, photon blockade^23–25^ including weak force sensing^26,27^. Furthermore, the bipartite entanglement between the cavity field and the mechanical oscillator in the Fabry-Perot cavity was reported in seminal work^28^ whereas Paternostro et al also proposed a scheme that showed the signatures of multipartite entanglement generated by radiation pressure in a cavity optomechanical system^29^. So, the quantum entanglement in cavity optomechanical systems has major practical implications for quantum information processing and quantum technologies^30^. It also provides a robust platform for studying the boundary between classical and quantum physics, as well as exploring the interface between quantum mechanics and the macroscopic world^31^. At the same time, another important quantum correlation known as quantum coherence^17,32,33^ which arises due to the well-known superposition principle is also a key concept in quantum information, quantum thermodynamics and quantum optics^34–37^. Based on a rigorous mathematical framework to quantify it^38^, the macroscopic quantum coherence in a simple cavity optomechanical system was first studied in^16^ and later on also explored in hybrid optomechanical systems^39^, a whispering-gallery-mode optical microresonator^40^ as well as the transfer of quantum coherence between the cavity and mechanical modes in a linear optomechanical system^41^.

In all of these above mentioned works, the radiation pressure induces the optomechanical interaction of the mechanical mode with the cavity field due to the exchange of linear momentum in between these two modes. However, Bhattacharya and Meystre^42^ first proposed a rotational-cavity optomechanical system in which a macroscopic rotating mirror is coupled to a Laguerre–Gaussian (L–G) cavity mode only through the exchange of orbital angular momentum and later on subsequently the ground-state cooling of the rotating mirror due to the action of the radiation torque in a double L–G cavity with an atomic ensemble studied in Ref.^43^. All these works on the rotating mirror lead to further investigation of macroscopic quantum phenomena in rotational cavity optomechanical systems such as bipartite quantum entanglement^42,44–49^,macroscopic quantum coherence^32^, OMIT phenomena^50–53^ including its applications for the measurement of orbital angular momentum^50,54,55^.

Overall, hybrid quantum systems have the potential to unlock new capabilities in the field of quantum technology^56^. These interfaces are generally composed of different quantum systems and simultaneously can perform several tasks like reliable storage, processing and transmission of information^57^. Seminal progress in the direction of COM leads to the design of such hybrid quantum systems that can be used to investigate coherent dynamics simultaneously both at the microscopic and macroscopic domains for example optomechanical system with atomic gas^58,59^, Bose-Einstein condensate^60^ including semiconductor nanostructures^61^. One of the major advantages of these spin ensembles is to significantly enhance the bipartite entanglement in COM as found in^62,63^. At present, macroscopic ferromagnetic materials such as yttrium iron garnet (YIG) crystal attracted significant attention due to its high-quality magnetic properties^64,65^. The collective excitation of spins in YIG spheres known as ”magnons” is a promising platform for developing a robust macroscopic quantum interface as its frequency is effectively controlled by adjusting a bias magnetic field^64–67^. The spin density in magnons is also significantly higher than other spin ensembles such as two level atomic ensembles and hence makes it possible to realise strong coupling with the cavity field^64–66,68^. In addition to this, the Kittel mode existing inside the YIG sphere also has unique characteristics such as a low damping rate and a long coherence time^69–71^. So far, various interesting quantum phenomena such as tunable magnomechanically induced transparency and absorption^66,72^, Four wave mixing^73^, Magnon Kerr effect^74–76^, bipartite and tripartite entanglement^77–88^ including nonclassical quantum correlations^33,89–91^ successfully explored in cavity magnomechanical systems. Moreover, Xiong et al recently proposed a theoretical scheme to realize the nonreciprocal bipartite and tripartite entanglements among magnons, photons, and phonons in a hybrid cavity-magnon optomechanical system^92^.

Based on these studies, in this present work, we have investigated the bipartite entanglement and quantum coherence in the hybrid L–G rotational cavity optomechanical system coupled with two magnon modes. We explore in detail the effect of various physical parameters on bipartite entanglement and quantum coherence. We also analyze the underlying physical mechanisms in detail and elucidate the difference between quantum entanglement and coherence.

This paper is organized as follows. In Sect. "The model Hamiltonian", we introduce the model Hamiltonian for the L–G rotational cavity optomechanical system coupled with two magnon modes. Section "Quantum dynamics" deals with the quantum Langevin equations as well as their steady-state solutions. In this Section, we also calculate the quadrature fluctuation equations for our system Hamiltonian. In Sect. "Bipartite entanglement and macroscopic quantum coherence", we provide an analytical mathematical formulation for exploring bipartite entanglement and macroscopic quantum coherence between different modes. We discuss the effects of various physical parameters on bipartite entanglement and macroscopic quantum coherence in Sect. "Results and discussion". We conclude our results in Sect. "Conclusion".

## The model Hamiltonian

**Figure 1:**
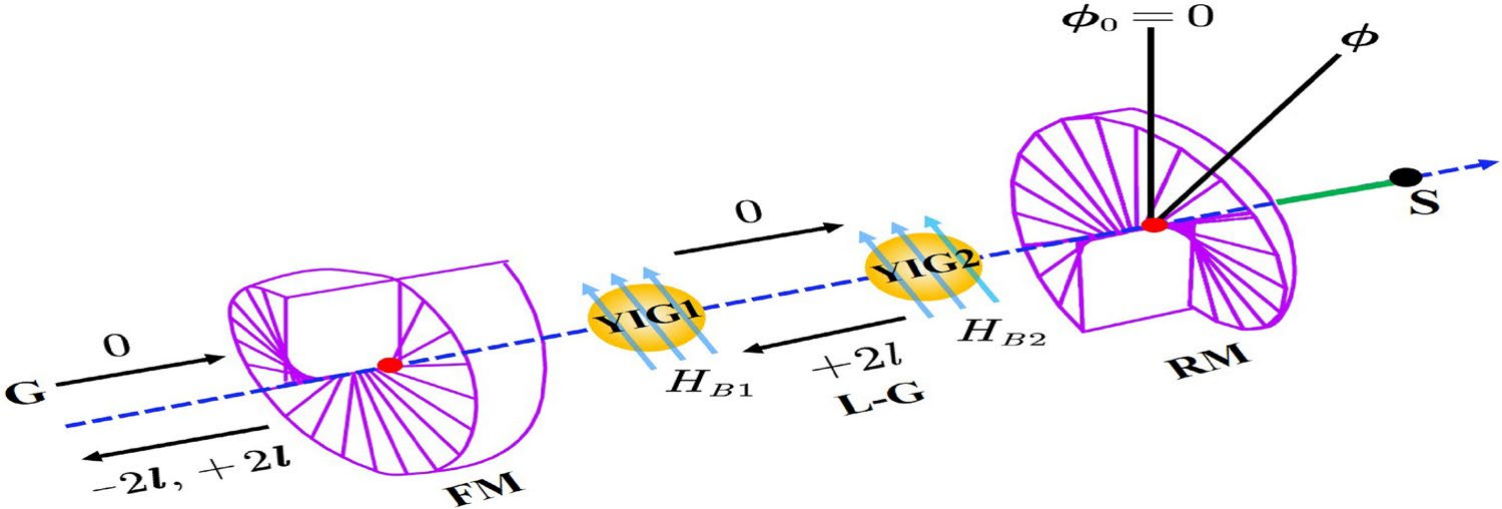
Schematic diagram of an L–G rotational-cavity optomechanical setup containing a single RM and two YIG spheres inside it. The equilibrium position of RM is given as $\phi _0$ whereas the angular displacement is represented by the angle $\phi$. At the same time, the interaction between both the magnon modes and the L–G cavity mode occurs respectively due to the presence of uniform bias magnetic fields $H_{B_1}$ and $H_{B_2}$. A Gaussian beam of frequency $\omega _{l}$ also externally drives the cavity mode.

As depicted in Fig. 1, the L–G rotating cavity optomechanical system is composed of a fixed mirror (FM) and a rotational mirror (RM) mounted on support as well as which can rotate about the cavity axis. Both the mirrors have spiral phase elements and the FM is partially transparent however it does not change the topological charge of any beam which passes through it. However, it removes a fixed topological charge of an incident beam 2*l* upon reflection. The RM is perfectly reflective and adds a charge of 2*l* to a beam reflected from it. When a Gaussian input beam passes through this FM, the reflected component gets a topological charge $-2l$ whereas the transmitted one has a charge 0. The transmitted beam with charge 0 gets reflected again from the RM and charged to 2*l*. When it returns back subsequent reflection at FM results in a mode with charge 0 whereas the transmission component comes with a charge 2*l*^32,42,46^. This system also includes two YIG spheres which give two magnon modes excited by bias magnetic fields $H_{B_1}$ and $H_{B_2}$. Our system Hamiltonian reads as^92^:

1 $$\begin{aligned} \mathcal {H}/\hbar= & \, \omega _{{a}}a^{\dagger }a+\omega _{{m} _{1}}m_{1}^{\dagger }m_{1}+ \omega _{{m}_{2}}m_{2}^{\dagger }m_{2}+ \frac{1}{2} \omega _{\phi }\left( L_{{z}}^{2}+\phi ^{2}\right) + g_{{m}_{1}a}\left( a+a^{\dagger }\right) \left( m_{1}+m_{1}^{\dagger }\right) \nonumber \\ {}{} & {} + g_{{m}_{2}a}\left( a+a^{\dagger }\right) \left( m_{2}+m_{2}^{\dagger }\right) - g_{\phi \text {a}}a^{\dagger }a\phi +i \mathcal {E}\left( a^{\dagger }e^{-i\omega _{l}t}-ae^{i\omega _{l}t}\right) \end{aligned}$$

where *a* and $a^{\dagger }$ (with commutation relation $[{a},{ a^{\dagger }}] =1$) are the annihilation and creation operators of the L–G cavity mode with frequency $\omega _{a}$. Similarly, $m_{j}$ and $m^{\dagger }_{j}$ ($[m_{j},{ m_{k}^{\dagger }}] =\delta _{jk}$) represents the annihilation and creation operators of the *j*th magnon mode with frequency $\omega _{m_j}$ determined by the gyro-magnetic ratio $\gamma$ and the bias magnetic field $H_{B_j}$ related through $\omega _{m_j}=\gamma H_{B_j}$ where ($j=1,2$). The other quantum operators $L_{z}$ and $\phi$ describe the angular momentum and angular displacement of the RM respectively with corresponding commutation relation $[L_{z},\phi ]=-i$ and $\omega _{\phi }$ is its angular frequency. Here we would like to mention that the RM in this system is modeled as a harmonic oscillator for the angular deviations $\phi \ll 2\pi$ which has the equilibrium position $\phi _0=0$. The coupling rate $g_{{m}_{j}}$ denotes the linear coupling between the L–G cavity mode and the *j*th magnon mode whereas the term $g_{\phi {a}}$ represents the optorotational coupling rate with relation $g_{\phi {a}}=(cl/L)\sqrt{\hbar /I\omega _{\phi }}$. Here, *L* is the length of the cavity and $I=m R^{2}/2$ is the moment of inertia of the RM of mass *m* and radius *R* about the cavity axis. The last term describes the input driving by a Gaussian beam with frequency $\omega _{l}$ where $\mathcal {E}$ is related to input laser power $P_{l}$ as $\mathcal {E} = \sqrt{{2\gamma _{a} P_{l}/{\hbar \omega _{l}}}}$.

## Quantum dynamics

To examine the quantum dynamics of this system Hamiltonian, we exploit the well-known quantum Langevin equations (QLEs), which take into account the Brownian noise acting on the rotating mirror as well as the vacuum fluctuations entering the L–G cavity.

In the frame rotating at the driving laser frequency $\omega _{l}$ as well as applying the rotating-wave approximation, the corresponding QLEs can be written as:

2 $$\begin{aligned} \dot{\phi }= & \, \omega _{\phi }L_{{z}}, \nonumber \\ \dot{L}_{z}= & {} -\omega _{\phi }\phi -\frac{D_{\phi }}{I}L_{{z}}+g_{\phi {a}}a^{\dagger }a+\xi ^{{in}}, \nonumber \\ \dot{a}= & {} -\left( \gamma _{a}+ i\Delta _{a}\right) a-ig_{{m}_{1}}m_{1}-ig_{{m}_{2}}m_{2}+ig_{\phi {a}}\phi + \mathcal {E} + \sqrt{2\gamma _{{a}}}a^{{in}}, \nonumber \\ \dot{m}_{1}= & {} -\left( \gamma _{{m}_{1}}+i \Delta _{{m}_{1}}\right) m_{1}-ig_{{m}_{1}}a+\sqrt{2\gamma _{{m}_{1}}}m_{1}^{{in}}, \nonumber \\ \dot{m}_{2}= & {} -\left( \gamma _{{m}_{2}}+i\Delta _{{m}_{2}}\right) m_{2}-ig_{{m}_{2}}a+\sqrt{2\gamma _{{m}_{2}}}m_{2}^{{in}}. \end{aligned}$$

Here $\Delta _{a}=\omega _{a}-\omega _{l}$, $\Delta _{{m}_{1}} =\omega _{{m}_{1}}-\omega _{l}$ and $\Delta _{{m}_{2}} =\omega _{{m}_{2}}-\omega _{l}$ denote the detuning of the cavity photon and both the magnon modes respectively with respect to the external driving field with frequency $\omega _{l}$. In addition, $D_{\phi }$ represents the intrinsic damping constant of the RM whereas the $\gamma _{{a}}$ is the decay rate of the L–G cavity field and the $\gamma _{{m}_{j} }$ is the $j^{th}$ magnon mode decay rate. The terms containing $a^{{in}}$ and $m_{j}^{{in}}$ are the noise operators for the cavity and $j^{\text {th}}$ magnon modes respectively, whereas $\xi ^{\text {in}}$ is the Brownian noise operator which represents the mechanical noise that couples to the RM from its environment. The mean values of these noise operators are zero, however, their nonzero correlation functions are given as^27,32,93–95^:

3 $$\begin{aligned} \left\langle a^{{in}}\left( t\right) a^{{in},\dagger }( t^{^{\prime }}) \right\rangle= & \, \left( 2\bar{n}_a+1\right) \delta ( t-t^{^{\prime }}), \nonumber \\ \left\langle m_{1}^{{in}}\left( t\right) m_{1}^{{in},\dagger }( t^{^{\prime }}) \right\rangle= & \, \left\langle m_{2}^{{in} }\left( t\right) m_{2}^{{in},\dagger }( t^{^{\prime }}) \right\rangle =\left( 2\bar{n}_m+1\right) \delta ( t-t^{^{\prime }}), \nonumber \\ \left\langle \xi ^{in}(t)\xi ^{in}(t^{^{\prime }})\right\rangle= & \, \dfrac{D_{\phi }}{I}\left( 2\bar{n}_{\phi }+1\right) \delta (t-t^{^{\prime }}). \end{aligned}$$

In Eqs. (3), the average thermal photon, magnon and phonon numbers at temperature *T* are given by $\bar{n}_o=\left[ e^{\hbar \omega _{o}/k_{B}T}-1\right] ^{-1}$ ($o=a,m_{1}, m_{2}$) whereas $\bar{n}_{\phi }=\left[ e^{\hbar \omega _{eff}/k_{B}T}-1\right] ^{-1}$ and $\omega _{eff}$ denotes the effective rotation frequency of the RM given as^32^.

4 $$\begin{aligned} \omega _\text {eff}^{2}\approx & {} \omega _{\phi }^{2}-\dfrac{2\xi _{\phi }^{2}\gamma _a P_{in}}{ I\omega _{a}}\left( \frac{\Delta _{a} }{\Delta _{a} ^{2}+\left( {\gamma _a }/{2} \right) ^{2}}\right) \times \frac{\left( {\gamma _a }/{2}\right) ^2 -\left( \omega ^{2}-\Delta _{a} ^{2}\right) }{\left[ \left( { \gamma _a }/{2}\right) ^{2}+\left( \omega -\Delta _{a} \right) ^{2}\right] \left[ \left( {\gamma _a }/{2}\right) ^{2}+\left( \omega +\Delta _{a} \right) ^{2} \right] }. \end{aligned}$$

### Steady state

The steady-state response of the RM, L–G cavity mode and both the magnon modes under the limit of long time are obtained as:

5 $$\begin{aligned} L_{{z,s}}= & \, 0, \nonumber \\ \phi _{{s}}= & \, \frac{g_{\phi {a}}\left| a_{{s}}\right| ^{2}}{\omega _{\phi }}, \nonumber \\ m_{1,{s}}= & \, \frac{-ig_{{m}_{1}}a_{{s}}}{\gamma _{{m}_{1}}+i\Delta _{{m}_{1}}}, \nonumber \\ m_{2,{s}}= & \, \frac{-ig_{{m}_{2}}a_{{s}}}{\gamma _{{m}_{2}}+i\Delta _{{m}_{2}}}, \nonumber \\ a_{\text {s}}= & \, \frac{\mathcal {E}}{\gamma _{a} + i\Delta _{{a}}^{\prime }+(\lambda _{1}+\lambda _{2})}, \end{aligned}$$

with the effective cavity detuning $\Delta _{{a}}^{\prime }= \Delta _{{a}}- g_{\phi _{a}}\phi _{{s}}$ whereas $\lambda _{1}=\frac{g_{{m}_{1}}^{2}}{\gamma _{{m}_{1}}+i\Delta _{{m}_{1}}}$ and $\lambda _{2}=\frac{g_{\text {m}_{2}}^{2}}{\gamma _{{m}_{2}}+i\Delta _{\text {m}_{2}} }$.

### Quantum fluctuations

To study the influence of quantum fluctuations on the evolution of system dynamics, we decompose each operator present into Eq. (2) into a sum of its steady state value and a small quantum fluctuation operator, *i.e.* $o=o_s+\delta o$ with $o=a, m_{1}, m_{2},L_{\text {z}}, \phi$. We can also neglect the nonlinear terms as the mean value of the physical quantity is much larger than its fluctuation and the linearized QLEs for the fluctuations can therefore straightforwardly is written in the following compact form:

6 $$\begin{aligned} \dot{u}(t)=\mathcal {A}u(t)+\eta (t). \end{aligned}$$

Here $u^{\textrm{T}}(t)=\left[ \delta \phi (t), \delta L_{z}(t), \delta X(t), \delta Y(t), \delta x_{1}(t), \delta y_{1}(t), \right. \left. \delta x_{2}(t), \delta y_{2}(t)\right]$ is the vector of quadrature fluctuations with $\delta X =(\delta a+\delta a^{\dagger })/\sqrt{2}$, $\delta Y=(\delta a- \delta a^{\dagger })/i \sqrt{2}$, $\delta x_{j} =(\delta m_{j}+ \delta m_{j}^{\dagger })/\sqrt{2}$ and $\delta y_{j} =(\delta m_{j}- \delta m_{j}^{\dagger })/i\sqrt{2}$ ($j=1,2$). $\eta ^{\textrm{T}}(t)=\left[ 0,\xi ^{{in}},\sqrt{ 2\gamma _{{a}}}X^{{in}}, \sqrt{ 2\gamma _{{a}}}Y^{{in}}, \sqrt{2\gamma _{{m}_{1}}}x_{1}^{{in}},\sqrt{2\gamma _{{m}_{1}}}y_{1}^{{in}}, \right. \left. \sqrt{2\gamma _{{m}_{2}}}x_{2}^{{in}},\sqrt{2\gamma _{{m}_{2}}}y_{2}^{{in}}\right]$ is the input noises vector with $X ^{{in}}=( a^{{in}}+ a^{in,\dagger })/\sqrt{2}$, $Y^{in}=( a^{in}- a^{{in},\dagger })/i \sqrt{2}$, $x^{in}_{j} =( m^{in}_{j}+ m_{j}^{{in},\dagger })/\sqrt{2}$ and $y^{in}_{j} =( m^{in}_{j}- m_{j}^{{in},\dagger })/i\sqrt{2}$ ($j=1,2$). The drift matrix $\mathcal {A}$ for this system is given by:

7 $$\begin{aligned} \mathcal {A}= \begin{pmatrix} 0 &{} \quad \omega _{\phi } &{} \quad 0 &{} \quad 0 &{} \quad 0 &{} \quad 0 &{} \quad 0 &{} \quad 0 \\ -\omega _{\phi } &{} \quad -\gamma _{\phi } &{} \quad G_{\phi {a}} &{} \quad 0 &{} \quad 0 &{} \quad 0 &{} \quad 0 &{} \quad 0 \\ 0 &{} \quad 0 &{} \quad -\gamma _{{a}} &{} \quad \Delta _{{a}}^{\prime } &{} \quad 0 &{} \quad g_{{m} _{1}} &{} \quad 0 &{} \quad g_{{m}_{2}} \\ G_{\phi {a}} &{} \quad 0 &{} \quad -\Delta _{{a}}^{\prime } &{} \quad -\gamma _{{a}} &{} \quad -g_{{m}_{1}} &{} \quad 0 &{} \quad -g_{\text {m}_{2}} &{} \quad 0 \\ 0 &{} \quad 0 &{} \quad 0 &{} \quad g_{{m}_{1}} &{} \quad -\gamma _{{m}_{1}} &{} \quad \Delta _{{m} _{1}} &{} \quad 0 &{} \quad 0 \\ 0 &{} \quad 0 &{} \quad -g_{{m}_{1}} &{} \quad 0 &{} \quad -\Delta _{{m}_{1}} &{} \quad -\gamma _{{m} _{1}} &{} \quad 0 &{} \quad 0 \\ 0 &{} \quad 0 &{} \quad 0 &{} \quad g_{{m}_{2}} &{} \quad 0 &{} \quad 0 &{} \quad -\gamma _{{m}_{2}} &{} \quad \Delta _{ {m}_{2}} \\ 0 &{} \quad 0 &{} \quad -g_{{m}_{2}} &{} \quad 0 &{} \quad 0 &{} \quad 0 &{} \quad -\Delta _{{m}_{2}} &{} \quad -\gamma _{ {m}_{2}} \end{pmatrix}, \end{aligned}$$

where $G_{\phi {a}}=\sqrt{2}g_{\phi \text {a}}a_{\text {s}}$ represents the effective optorotational coupling parameter.

## Bipartite entanglement and macroscopic quantum coherence

As the system Hamiltonian is of Gaussian nature, its state can be fully described in the stationary regime by the $8\times 8$ covariance matrix (CM) of elements given as $V_{jk}(\infty )=\langle \left[ u_j(\infty ),u_k(\infty )\right] _+\rangle /2$, which is the solution of the following standard Lyapunov equation:

8 $$\begin{aligned} \mathcal {A}\,V+ V \mathcal {A}^T+D=0, \end{aligned}$$

where *D* is the diffusion matrix describing the stationary noise correlations. It is defined by $D_{jk}\,\delta (t-t^{\prime })=\langle \left[ \eta _j(t),\eta _k(t^{\prime })\right] _+\rangle /2$ and determined by using the correlation functions of Eq. (3) as $D=\textrm{diag}[0,\gamma _{\phi }(2\bar{n}_{\phi }+1),\gamma _a(2\bar{n}_{a }+1),\gamma _a(2\bar{n}_{a }+1),\gamma _{{m}_{1}}(2\bar{n}_{m_1 }+1),\gamma _{{m}_{1}}(2\bar{n}_{m_1 }+1),\gamma _{{m}_{2}}(2\bar{n}_{m_2 }+1),\gamma _{{m}_{2}}(2\bar{n}_{m_2 }+1)]$.

Furthermore, as the analytical solutions for the Eq. (8) is very complex, we can employ numerical simulations to investigate the bipartite entanglement and quantum coherence of this proposed system. This solution can be presented as:

9 $$\begin{aligned} V=\left( \begin{array}{cccc} V_{\phi } &{} \quad W_{\phi a} &{} \quad W_{\phi m_1} &{} \quad W_{\phi m_2} \\ W_{\phi a}^{\textrm{T}} &{} \quad V_{a} &{} \quad W_{a m_1} &{} \quad W_{a m_2} \\ W_{\phi m_1}^{\textrm{T}} &{} \quad W_{a m_1}^{\textrm{T}} &{} \quad V_{m_1} &{} \quad W_{m_1 m_2} \\ W_{\phi m_2}^{\textrm{T}} &{} \quad W_{a m_2}^{\textrm{T}} &{} \quad W_{m_1 m_2}^{\textrm{T}} &{} \quad V_{m_2} \end{array}\right) , \end{aligned}$$

where $V_{j}$ ($j=\phi ,a,m_1,m_2$) is the $2\times 2$ matrix representing the local properties of the rotating mirror, L–G cavity mode and magnon modes. $W_{jk}$ ($j,k=\phi ,a,m_1,m_2$) is the $2\times 2$ matrix describing the correlations between the corresponding modes.

In order to explore the entanglement between different bipartitions, i.e., the L–G cavity mode and phonon mode, the magnon mode ($m_1$) and phonon mode, the L–G cavity mode and magnon mode ($m_1$) as well as in between the magnon mode ($m_1$) and magnon mode ($m_2$), we employ logarithmic negativity ($E_N$) as a measure of bipartite entanglement defined as^95–97^:

10 $$\begin{aligned} E_{N}=\max \left[ 0,-\ln 2\vartheta ^{-}\right] , \end{aligned}$$

where $\vartheta ^{-}=2^{-1/2}\left[ \Xi -\sqrt{\Xi ^{2}-4\det V_{jk}}\right] ^{1/2},$ with $\Xi =\det V_{j} +\det V_{k} -2\det W_{jk}$ ($j\ne k=\phi ,a,m_{1},m_{2}$). Here $V_{jk}$ is a $4\times 4$ submatrix of the correlation matrix *V* that captures the pairwise entanglement between two interesting modes, it can be rewritten as:

11 $$\begin{aligned} V_{jk}= \begin{pmatrix} V_{j} &{} \quad W_{jk} \\ W_{jk} ^{\text {T}} &{} \quad V_{k} \end{pmatrix} , \end{aligned}$$

Furthermore, We now provide a mathematical formulation of the quantum coherence between various Gaussian modes. Generally, the quantification of quantum coherence in a given one-mode Gaussian state $\rho (V,\overrightarrow{d})$ can be determined by considering the covariance matrix and the mean value vector, as follows^98^:

12 $$C\left[ {\rho \left( {V,\overrightarrow{d}} \right)} \right] = f\left( {2n + 1} \right) - f\left( \vartheta \right),$$

where $\vartheta =\sqrt{\det V}$ is the symplectic eigenvalue of *V* and

13 $$\begin{aligned} f(x)= & {} \frac{x+1}{2}\log _{2}(\frac{x+1}{2})-\frac{x-1}{2}\log _{2}(\frac{ x-1}{2}), \end{aligned}$$

14 $$\begin{aligned} {n}= & {} \left( \textrm{tr} (V)+d_{1}^{2}+d_{2}^{2}-2\right) /4. \end{aligned}$$

The above result can be easily generalized to the multimode Gaussian state, but for our discussion, we will only consider the two-mode Gaussian state. Here our main focus is on calculating the quantum coherence of a two-mode Gaussian state, which can be determined using the following expression^32,98^:

15 $$C^{{jk}} \left[ {\rho \left( {V_{{jk}} ,\overrightarrow{d}} \right)} \right] = \sum\limits_{{\mu = j,k}} f \left( {2n_{\mu } + 1} \right) - \sum\limits_{{i = \pm }} f \left( {\vartheta _{{jk,i}} } \right),$$

where the two symplectic eigenvalues of $V_{jk}$ are $\vartheta _{jk,\pm }=2^{-1/2}\left[ \Xi _{jk} \pm \sqrt{\Xi _{jk}^{2}-4\det V_{jk}} \right] ^{1/2}$ with $\Xi _{jk} =\det V_{j} +\det V_{k} +2\det W_{jk}$.

Therefore, it is possible to compute different types of two-mode quantum entanglement and quantum coherence by employing Eqs. (10) and (15), and these calculations will be presented in the following section.

## Results and discussion

In this section, we will discuss the generation of the bipartite entanglement and the quantum coherence between the different bipartitions present in our system Hamiltonian. We have taken into account the parameters for the L–G cavity that can be easily achieved in the experiments^32,42–45,47,73,76^ and are given below,

$m=5$ ng, $R=10$ $\mu m$, $l=50$, $P_{l}=50$ mW, the laser wavelength $\lambda _l =810$ $\mu m$, the optical finesse $F=1.1\times 10^4$, the quality factor $Q=10^5$, $L=1$ mm, $\gamma _a/2\pi =0.5$ MHz, $\gamma _{m_1}/2\pi =\gamma _{m_2}/2\pi =3.75$ MHz, $\gamma _{\phi }/2\pi =100$ Hz, $g_{m_1}/2\pi =g_{m_2}/2\pi = 4.5$ MHz, $\omega _{\phi }/2\pi =21$ MHz and $T=0.4$ K.

At first, we analyse various bipartitions, namely, $E^{a\phi }_{N}$ ($C^{a\phi }$), $E^{m_1\phi }$ ($C^{m_1\phi }$), $E^{a m_1}_{N}$ ($C^{a m_1}$) and $E^{m_1m_2}_{N}$ ($C^{m_1m_2}$) which respectively denote the bipartite entanglement (quantum coherence) between L–G cavity mode and phonon mode; magnon mode ($m_1$) and phonon mode; L–G cavity mode and magnon mode ($m_1$) and finally in between magnon mode ($m_1$) and magnon mode ($m_2$).

**Figure 2:**
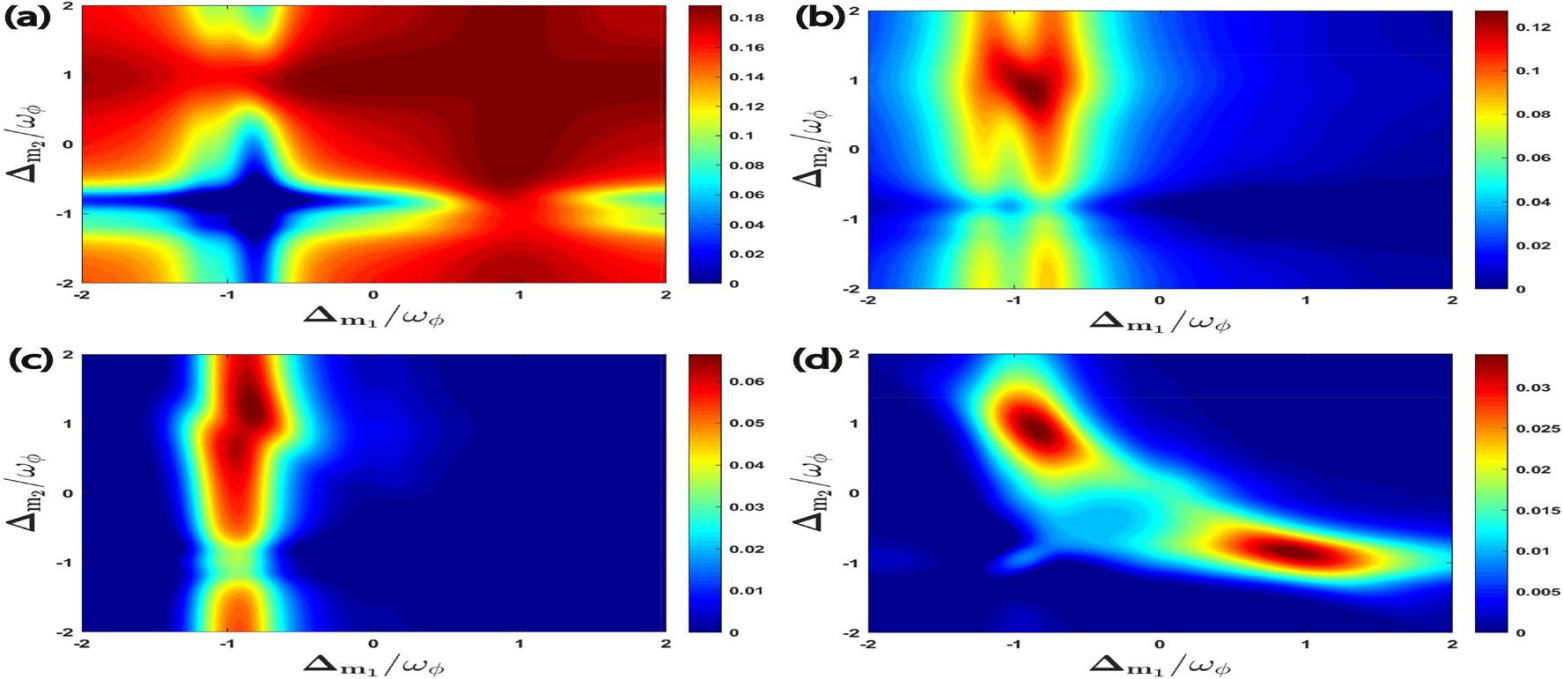
Density plot of (**a**) $E^{a\phi }_{N}$, (**b**) $E^{m_1\phi }_{N}$, (**c**) $E^{a m_1}_{N}$, (**d**) $E^{m_1m_2}_{N}$ versus the normalized detuning $\Delta _{m_1}/\omega _{\phi }$ and $\Delta _{m_2}/\omega _{\phi }$. Here we have taken effective cavity detuning $\Delta _a^{\prime }=\omega _{\phi }$ for all cases.

In Fig. 2, we represent the four bipartite entanglements as a function of the normalized detuning $\Delta _{m_1}/\omega _{\phi }$ and $\Delta _{m_2}/\omega _{\phi }$. Here, we have taken the detuning of the L–G cavity mode perfectly resonant with the blue sideband regime of the RM, i.e. $\Delta _{a}^{\prime }=\omega _{\phi }$ , which also corresponds to the anti-Stokes process. This leads to significant cooling of the RM and so it enhances the entanglement phenomena. In Fig. 2a, the entanglement $E_{N}^{a\phi }$ almost get saturated with a fixed value when the detunings of both the magnon modes $m_{1}$ ($m_{2}$) are resonant only with the blue sideband regime of the RM, i.e., $\Delta _{m_1}=\Delta _{m_2}=\omega _{\phi }$. However, if we gradually change both the magnon detuning towards the red sideband regime of the RM, i.e., $\Delta _{m_1}=\Delta _{m_2}\simeq -\omega _{\phi }$, the bipartite entanglement $E_{N}^{a\phi }$ almost get zero. This is due to the presence of Stokes processes caused by both the magnon modes in this regime, which ultimately leads to significant heating of the RM. Hence, we do not obtain any entanglement between the L–G cavity mode and the phonon mode of the RM. Furthermore, it can be seen that in Fig. 2b,c, the bipartite entanglements $E^{m_1\phi }_{N}$ and $E^{am_1}_{N}$ exhibit a maximum value when the detuning of the magnon mode $m_{1}$ and the magnon mode $m_{2}$ are respectively resonant with the red and the blue sideband regime of the RM, i.e., $\Delta _{m_1}=-\Delta _{m_2}\simeq -\omega _{\phi }$. This means that both of these bipartitions mainly get their maximum values when the first magnon leads to Stokes process whereas the second magnon enhances anti-Stokes phenomena inside the cavity. Moreover, when the detunings of both the magnon modes are always kept in resonance with two different RM sidebands regimes, i.e., when $\Delta _{m_1}=-\Delta _{m_2}\simeq \pm \omega _{\phi }$ the maximum degree of bipartite entanglement $E^{m_{1}m_{2}}_{N}$ is attained as shown in Fig. 2d , which shows that both anti-Stokes and Stokes processes leading to simultaneous cooling and heating of the RM are required inside the cavity to get maximum entanglement between both the magnon modes.

**Figure 3:**
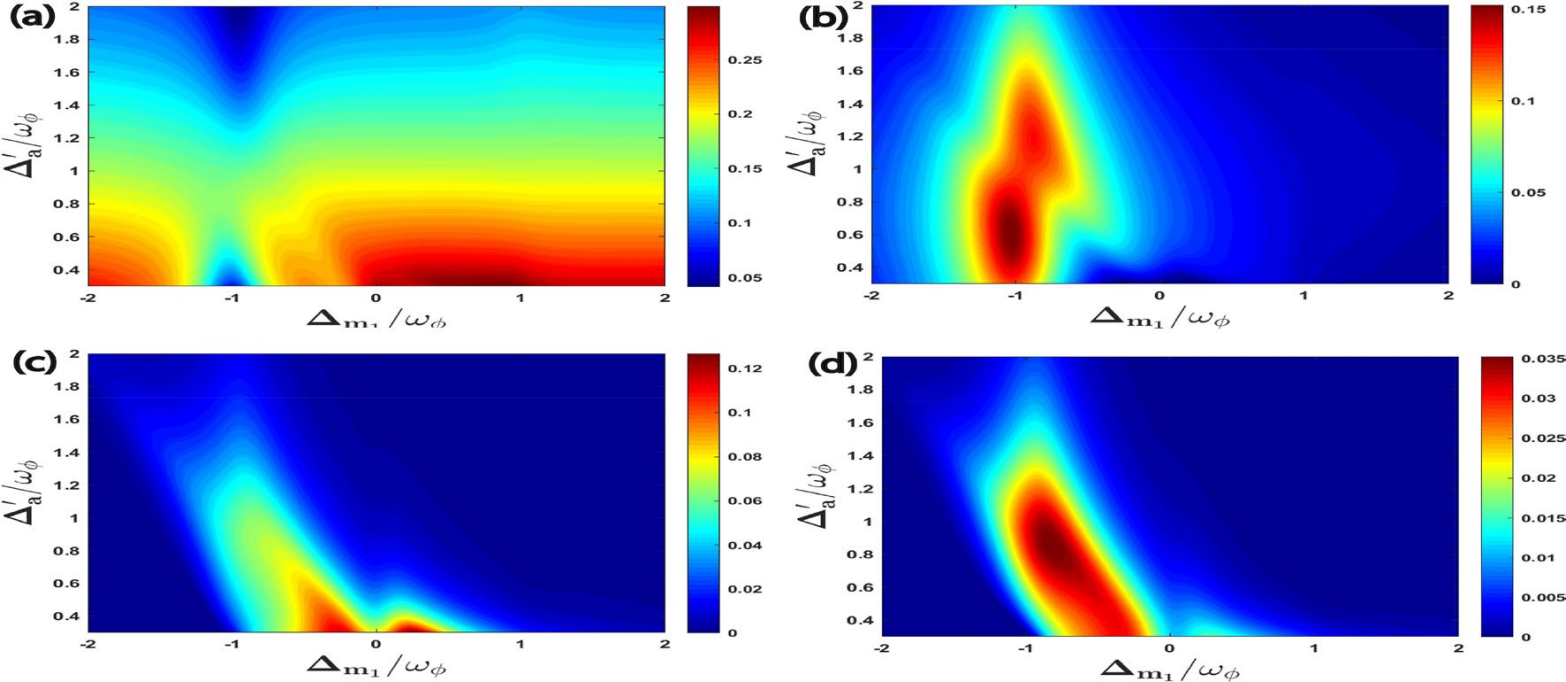
Density plot of (**a**) $E^{a\phi }_{N}$, (**b**) $E^{m_1\phi }_{N}$, (**c**) $E^{a m_1}_{N}$, (**d**) $E^{m_1m_2}_{N}$ versus the normalized detuning $\Delta _{m_1}/\omega _{\phi }$ and $\Delta _{a}^{\prime }/\omega _{\phi }$. In all four plots, second magnon detuning is $\Delta _{m_2}=\omega _{\phi }$. As shown in Fig. 2, all the four bipartite entanglements achieve higher values at this point.

In Fig. 3, for $\Delta _{m_2}=\omega _{\phi }$ we plot all the four bipartite entanglements as a function of the normalized detuning $\Delta _{m_1}/\omega _{\phi }$ and $\Delta _{a}^{\prime }/\omega _{\phi }$. We get a very strong bipartite entanglement $E^{a\phi }_{N}$ when the effective cavity detuning is at $\Delta _{a}^{\prime }\simeq 0.3\omega _{\phi }$ and the detuning of the magnon mode ($m_1$) is approximately resonant with the blue sideband of the RM, i.e. $\Delta _{m_1}=\omega _{\phi }$ , which leads to significant cooling of the RM and enhances $E^{a\phi }_{N}$ as shown in Fig. 3a. Moreover, Fig. 3b shows that the bipartite entanglement $E^{m_1\phi }_{N}$ reaches the maximum value when the detuning of the L–G cavity mode and the magnon detuning of first magnon ($m_1$) are respectively nearly resonant with the blue and the red sideband regime of the RM. This corresponds to simultaneous cooling and heating of the RM inside the cavity. As compared to other bipartions, $E^{am_1}_{N}$ achieves its maximum value with a very low value of effective cavity detuning as well as magnon detuning as shown in Fig. 3c. This implies that both anti-Stokes and Stokes processes of the RM should be suppressed inside the cavity. In addition, we can also see from Fig. 3d that bipartite entanglement $E^{m_{1}m_{2}}_{N}$ reaches its maximum value when the detunings due to L–G cavity mode and the magnon mode ($m_{1}$) are approximately resonant with the blue and the red sideband regime which corresponds to the simultaneous cooling and heating of the RM respectively.

**Figure 4:**
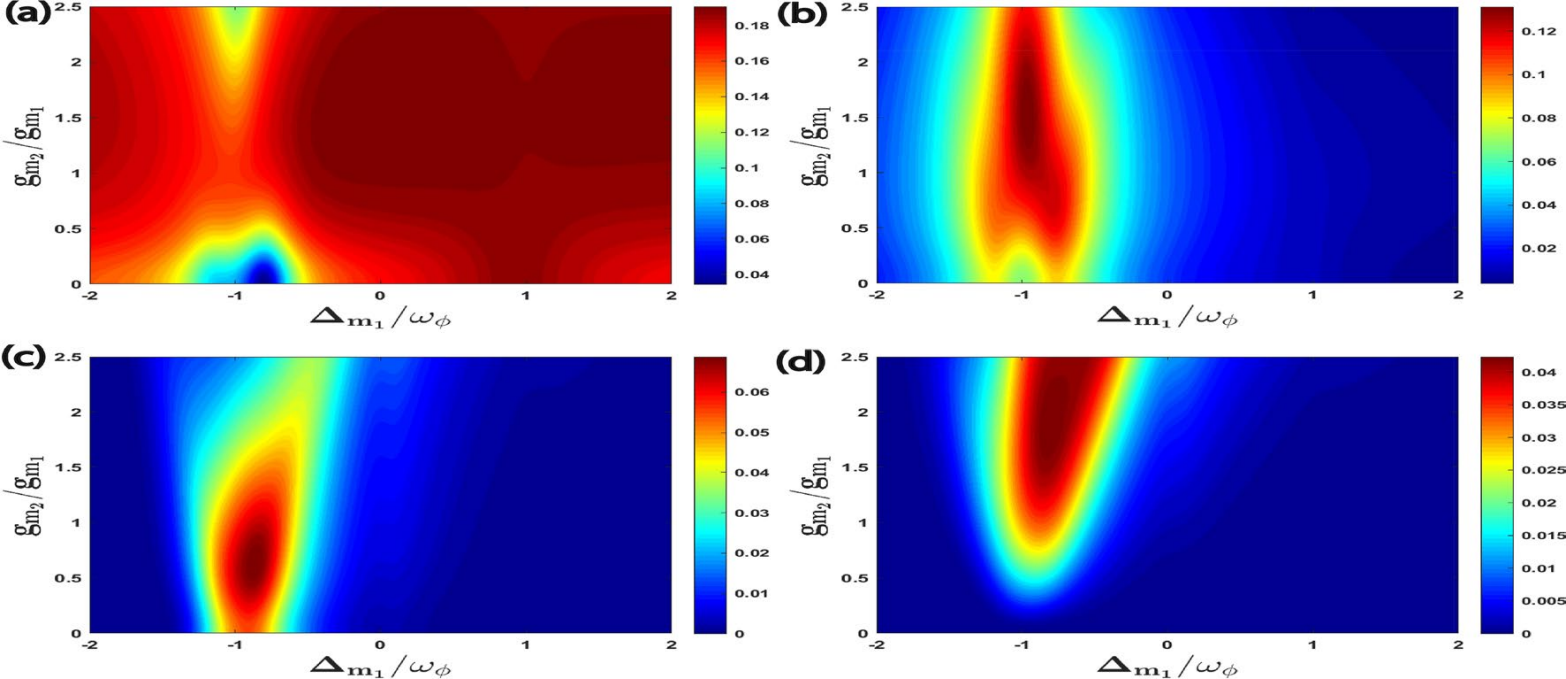
Density plot of (**a**) $E^{a\phi }_{N}$, (**b**) $E^{m_1\phi }_{N}$, (**c**) $E^{a m_1}_{N}$, (**d**) $E^{m_1m_2}_{N}$ versus the coupling strength $g_{m_2}/g_{m_1}$ and detuning $\Delta _{m_1}/\omega _{\phi }$. We consider other parameters as $\Delta _{m_2} = -0.9\omega _{\phi }$ and $\Delta _{a}^{\prime } = \omega _{\phi }$ in all four plots.

In Fig. 4, we have shown the four bipartite entanglements as a function of $\Delta _{m_1}/\omega _{\phi }$ and the coupling strength ratio $g_{m_2}/g_{m_1}$ where we have already taken $g_{m_1}/2\pi = 4.5$ MHz. It can be seen from Fig. 4 that there are different optimal couplings required to achieve maximum entanglement for each bipartition. In fact, for $E^{a\phi }_{N}$; $E^{m_1\phi }_{N}$; $E^{am_1}_{N}$ and $E^{m_1m_2}_{N}$, the optimal couplings are approximately $g_{m_2}\simeq g_{m_1}$; $g_{m_2}\simeq 1.5 g_{m_1}$; $g_{m_2}\simeq 0.7 g_{m_1}$ and $g_{m_2}\simeq 2 g_{m_1}$ respectively. It is important to mention here that there are no universal optimal coupling strength values of $g_{m_1}$ and $g_{m_2}$ that simultaneously maximize entanglement for all the possible bipartitions. This is due to the asymmetric transfer of entanglement caused by the interaction of different modes. So, the optimal coupling strengths depend upon the specific bipartition that we want to investigate and maximize in our system Hamiltonian. It can be also seen that when the detuning of the first magnon becomes resonant to the blue sideband regime of the RM, i.e. $\Delta _{m_1}= \omega _{\phi }$ then the bipartition $E^{a\phi }_{N}$ achieves its maximum value whereas all other three bipartitions become zero for a broader range of coupling strength ratio $g_{m_2}/g_{m_1}$. However, when the detuning of the first magnon is approximately resonant with the red sideband regime of the RM, which means that $\Delta _{m_1} \simeq -\omega _{\phi }$ then it leads to Stokes process and all the remaining three bipartitions can be controlled significantly with a proper choice of coupling strength ratio $g_{m_2}/g_{m_1}$. Therefore, the coupling strength ratio of both magnon modes plays an important role in controlling all the four bipartite entanglements in our proposed quantum system. Additionally, we can also note that although the maximum value of bipartite entanglement for directly coupled mode ($E^{a\phi }_{N}$) is the highest still indirectly coupled mode like $E^{m_1\phi }_{N}$ attains significant value in the current parameter regime. This result holds significant implications for the development of hybrid quantum systems mostly utilized in quantum information and quantum communication protocols. This is because optimizing bipartite entanglement for one subsystem may not necessarily result in optimal entanglement for other subsystems and different subsystems necessitate distinct coupling strength ratio to attain maximum entanglement.

**Figure 5:**
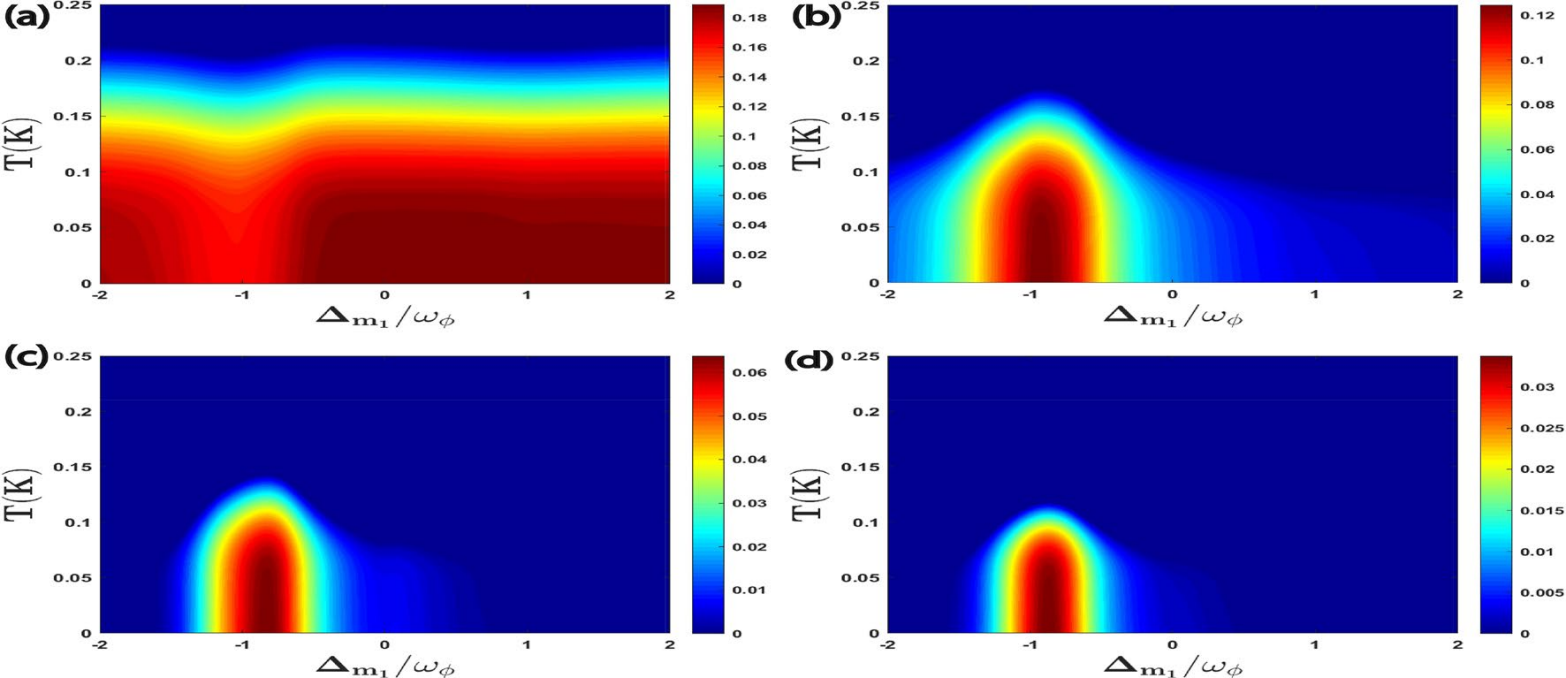
Density plot of (**a**) $E^{a\phi }_{N}$, (**b**) $E^{m_1\phi }_{N}$, (**c**) $E^{a m_1}_{N}$, (**d**) $E^{m_1m_2}_{N}$ versus the normalized detuning $\Delta _{m_1}/\omega _{\phi }$ and the temperature *T*. Other parameter is $\Delta _a^{\prime }=\Delta _{m_2}=\omega _{\phi }$ in all cases.

In Fig. 5, we give density plots of $E^{a\phi }_{N}$, $E^{m_1\phi }_{N}$, $E^{am_1}_{N}$, and $E^{m_1m_2}_{N}$ as a function of detuning $\Delta _{m_1}/\omega _{\phi }$ and temperature *T*. It can be seen that with a gradual change of magnon detuning $\Delta _{m_1}$, the effect of temperature *T* on all four bipartite entanglements can be significantly controlled. When $\Delta _{m_1}\simeq \omega _{\phi }$ (anti-Stokes process and leading to cooling of the RM), $E^{a\phi }_{N}$ has minimum thermal effects whereas for the other three bipartitions, it is observed around $\Delta _{m_1}\simeq -\omega _{\phi }$ (which leads to Stokes process and subsequently heating of the RM), however with a gradual increase in environment temperature *T* due to the decoherence phenomena, all the four bipartitions show degradation of bipartite entanglement in both red as well as blue sideband regimes of the RM.

**Figure 6:**
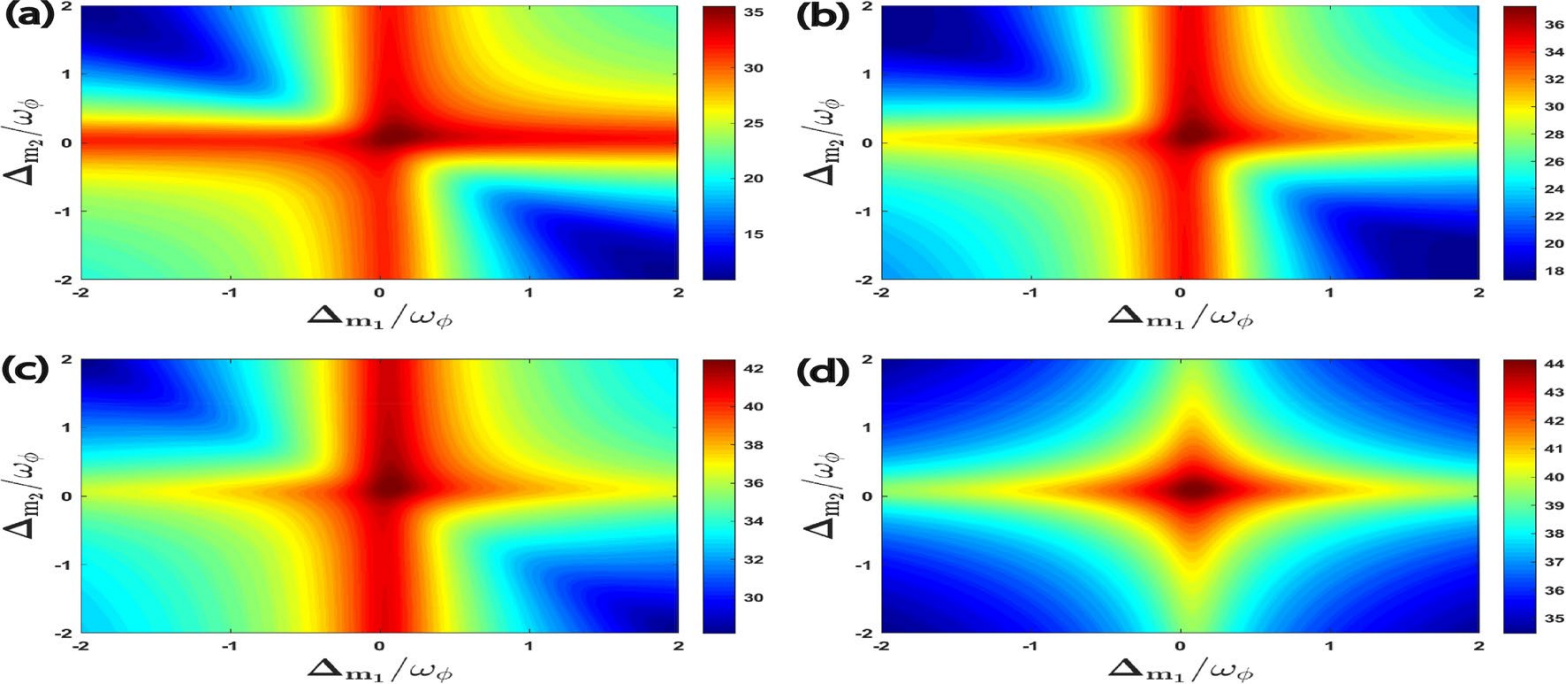
Density plot of (**a**) $C^{a\phi }$, (**b**) $C^{m_1\phi }$, (**c**) $C^{a m_1}$, (**d**) $C^{m_1m_2}$ versus the normalized detuning $\Delta _{m_1}/\omega _{\phi }$ and $\Delta _{m_2}/\omega _{\phi }$. We consider other parameter $\Delta _a^{\prime }=\omega _{\phi }$ in all four plots.

Furthermore, in Fig. 6, for an effective cavity detuning $\Delta _{a}^{\prime }=\omega _{\phi }$, we plot all the four types of quantum coherence, namely $C^{a\phi }$, $C^{m_1\phi }$, $C^{am_1}$ including $C^{m_1m_2}$ as a function of the normalized magnon detunings $\Delta _{m_1}/\omega _{\phi }$ and $\Delta _{m_2}/\omega _{\phi }$. As compared to the bipartite entanglement between different modes given in Fig. 2, the optimal detunings to achieve maximum quantum coherence between different bipartitions occur when both the magnon deunings are approximately resonant with the external cavity driving field, i.e., $\Delta _{m_1}=\Delta _{m_2}\simeq 0$. However, to enhance the various bipartite entanglements, it is necessary for both magnon modes to be detuned from the cavity driving field as shown in Fig. 2. The maximum values of quantum coherence achieved by each bipartition in this case is respectively given as $C^{a\phi }_{\max }\simeq 35.5$, $C^{m_1\phi }_{\max }\simeq 37.5$, $C^{am_1}_{\max }\simeq 42.5$, and $C^{m_1m_2}_{\max }\simeq 44$. We would like to mention here that the difference between the entanglement and the quantum coherence is that these two quantities measure completely different aspects of a given quantum system. Entanglement is a measure of the quantum correlation between different bipartitions present in the system. The maximum entanglement occurs when the two considered modes are maximally correlated for a particular set of parameters, e.g. Fig. 2. On the other hand, quantum coherence refers to the degree of the superposition between the different quantum states, and it can be related to the degree of coherence between different modes of the system. Each mode inside a given quantum system has its own quantum coherence, indicating its ability to exist in a superposition of states. This internal coherence always helps to maintain the system’s overall quantum coherence. The maximum of quantum coherence occurs when the system is in a state that is maximally superposed between different modes, which can be achieved in a different specific parameters regime, e.g. Fig. 6. Such quantum state also represents the ability of a given quantum system to exist in maximal superposition across multiple modes at the same time, demonstrating the complex interplay of quantum states inside the system. In this scenario, the dynamics of the quantum system have a distinct wave-like behavior, with each mode contributing coherently to the total quantum coherence.

**Figure 7:**
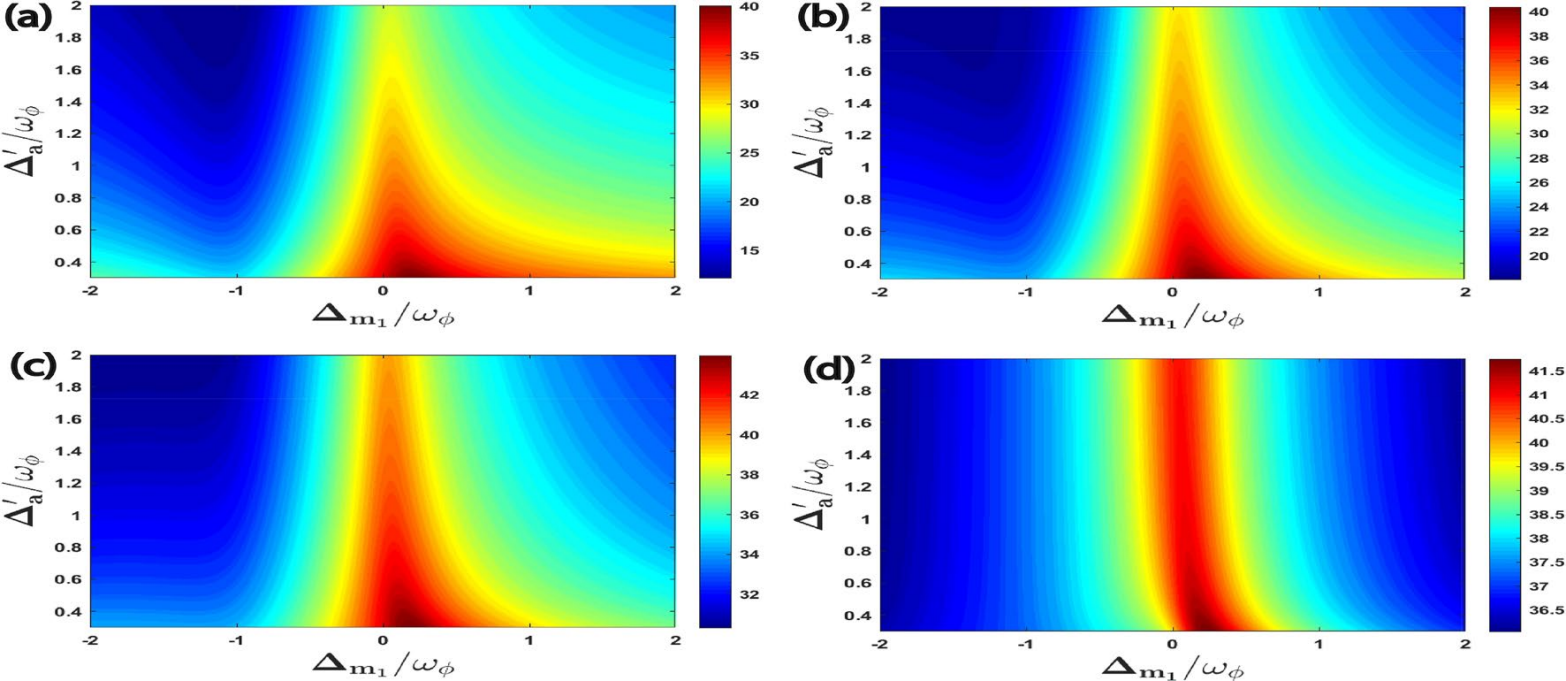
Density plot of (**a**) $C^{a\phi }$, (**b**) $C^{m_1\phi }$, (**c**) $C^{a m_1}$, (**d**) $C^{m_1m_2}$ versus the normalized detuning $\Delta _{m_1}/\omega _{\phi }$ and $\Delta _{a}^{\prime }/\omega _{\phi }$. We consider other parameter $\Delta _{m_2}=\omega _{\phi }$ in all four plots.

In Fig. 7, we have plotted all the four correlations of macroscopic quantum coherence as a function of the normalized first magnon detuning $\Delta _{m_1}/\omega _{\phi }$ and the effective cavity detuning $\Delta _{a}^{\prime }/\omega _{\phi }$ while keeping the second magnon detuning fixed at the blue sideband regime of the RM, which means $\Delta _{m_2}=\omega _{\phi }$. To achieve the maximum quantum coherence for all four correlations the magnon mode ($m_1$) should be approximately resonant with the external cavity driving field,i.e. $\Delta _{m_1} \simeq 0$, and the effective cavity detuning should be kept at $\Delta _{a}^{\prime }=0.3\omega _{\phi }$. It can be also seen that for the effective cavity detuning $\Delta _{a}^{\prime }=\omega _{\phi }$ which leads to the anti-Stokes process and subsequently cooling of the RM, all the four correlations of macroscopic quantum coherence gradually decrease although the value of $\Delta _{a}^{\prime }=\omega _{\phi }$ corresponds to RM cooling. Therefore, to achieve a higher degree of quantum coherence in this hybrid quantum system we should keep a smaller value of effective cavity detuning $\Delta _{a}^{\prime }$. This again shows that the optimal effectice cavity detuning to obtain maximum quantum coherence is completely different from those required for the bipartite entanglement. These results also highlight the importance of carefully selecting experimental parameters to achieve an efficient macroscopic quantum coherence phenomenon.

**Figure 8:**
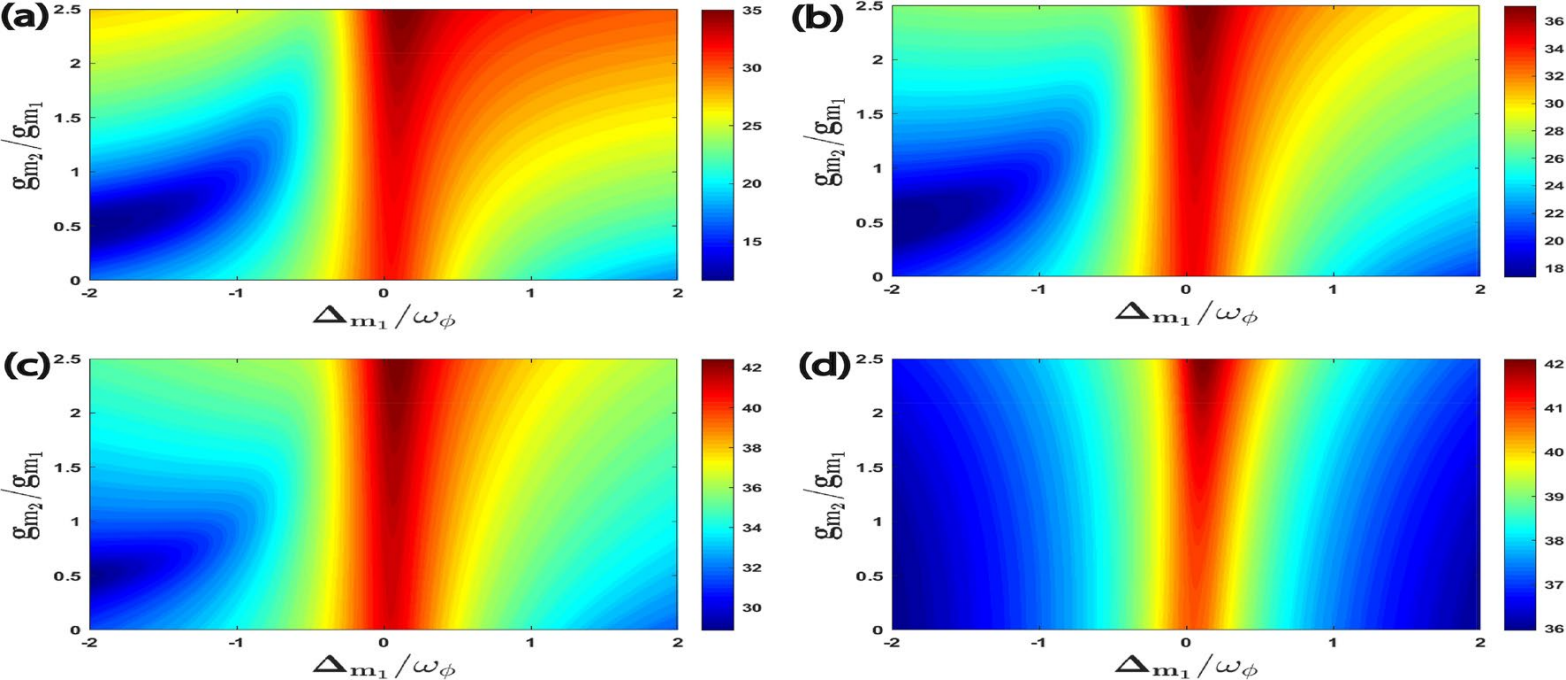
Density plot of (**a**) $C^{a\phi }$, (**b**) $C^{m_1\phi }$, (**c**) $C^{a m_1}$, (**d**) $C^{m_1m_2}$ versus the coupling strength $g_{m_2}/g_{m_1}$ and detuning $\Delta _{m_1}/\omega _{\phi }$. We take other parameters as $\Delta _{m_2}=-0.9\omega _{\phi }$ and $\Delta _{a}^{\prime }=\omega _{\phi }$ in all cases.

**Figure 9:**
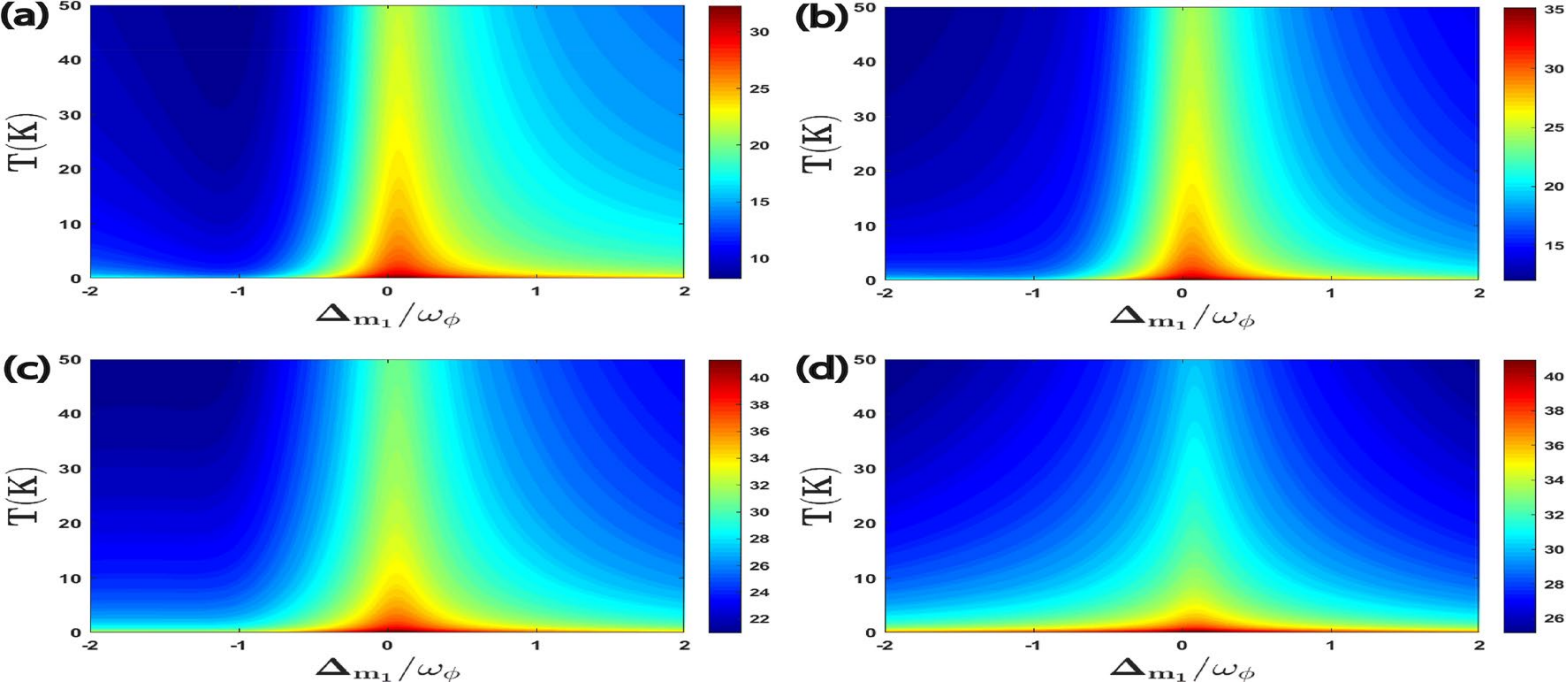
Density plot of (**a**) $C^{a\phi }$, (**b**) $C^{m_1\phi }$, (**c**) $C^{a m_1}$, (**d**) $C^{m_1m_2}$ versus the normalized detuning $\Delta _{m_1}/\omega _{\phi }$ and the temperature *T*. We take other parameters as $\Delta _a^{\prime }=\Delta _{m_2}=\omega _{\phi }$ in all four plots.

Further, we examine the effects of coupling ratio strength on quantum coherence as shown in Fig. 8. We plot all the four correlations for quantum coherence as a function of $\Delta _{m_1}/\omega _{\phi }$ and $g_{m_2}/g_{m_1}$ where we have already taken $g_{m_1}/2\pi = 4.5$ MHz. It can be seen that the maximum quantum coherence of all four bipartitons is achieved for coupling ratios strength $g_{m_2}\simeq 2.5 g_{m_1}$ and effective magnon detuning $\Delta _{m_1}\approx 0$. As compared to bipartite entanglement results given in Fig. 4 it is observed that we have just only one specific value for $g_{m_2}/g_{m_1}$ to enhance all the four correlations for quantum coherence. Furthermore, we plot all the four quantum coherence with varying first magnon detuning $\Delta _{m_1}/\omega _{\phi }$ and temperature *T* in Fig. 9. We can see that for magnon detuning $\Delta _{m_1}\approx 0$ whereas keeping $\Delta _a^{\prime }=\Delta _{m_2}=\omega _{\phi }$, all the four correlations persist despite thermal effects and have a significant amount of quantum coherence up to temperature 50 K. So, our proposed quantum system has a significant amount of quantum coherence even at higher temperature as compared to the bipartite entanglement given in Fig. 5, where due to the decoherence phenomena all the four bipartitions rapidly become zero with a gradual increase in environment temperature *T*. This is very important for the practical application of such systems in modern quantum technology however the optimal parameters for achieving significant quantum coherence are completely different from the bipartite entanglement.

## Conclusion

In conclusion, we have proposed a scheme to achieve maximum bipartite entanglement and quantum coherence in the hybrid L–G rotational optomechanical system containing two YIG magnetic nanospheres where both the YIG spheres are coupled to the L–G cavity mode through the magnetic dipole interaction. We theoretically investigate the variation of various bipartitions present in this quantum system for bipartite entanglement and macroscopic quantum coherence. We have also discussed in detail the parameters regime to achieve maximum bipartite entanglement and quantum coherence. We observed that the parameters set for achieving maximum bipartite entanglement are completely different from macroscopic quantum coherence. This is because one of them quantifies the correlation between different modes, while the other quantifies the degree of superposition of different quantum states. In addition, our proposed system has significant quantum coherence between different bipartitions even at higher temperatures. Our present results are insightful to understand as well as effectively control the various kinds of nonclassical quantum correlations in macroscopic quantum systems and have potential applications in quantum information, quantum metrology, and quantum computation.

## Data Availability

The datasets used and/or analyzed during the current study are available from the corresponding author on reasonable request.
